# KIR3DS1-Mediated Recognition of HLA-*B51: Modulation of KIR3DS1 Responsiveness by Self HLA-B Allotypes and Effect on NK Cell Licensing

**DOI:** 10.3389/fimmu.2017.00581

**Published:** 2017-05-26

**Authors:** Simona Carlomagno, Michela Falco, Maria Bono, Claudia Alicata, Lucia Garbarino, Michela Mazzocco, Lorenzo Moretta, Alessandro Moretta, Simona Sivori

**Affiliations:** ^1^Dipartimento di Medicina Sperimentale, Università degli Studi di Genova, Genova, Italy; ^2^Istituto Giannina Gaslini, Genova, Italy; ^3^Centro di Eccellenza per le Ricerche Biomediche, Università degli Studi di Genova, Genova, Italy; ^4^S.C. Laboratorio di Istocompatibilità e IBMDR, E.O. Ospedali Galliera, Genova, Italy; ^5^Dipartimento di Immunologia, IRCCS Ospedale Bambin Gesù, Roma, Italy

**Keywords:** human natural killer cells, activating killer-immunoglobulin-like receptors, KIR3DS1, natural killer cell education, HLA-B alleles, KIR/KIR-L interaction

## Abstract

Several studies described an association between killer-cell immunoglobulin-like receptor (KIR)/HLA gene combinations and clinical outcomes in various diseases. In particular, an important combined role for KIR3DS1 and HLA-B Bw4-I80 in controlling viral infections and a higher protection against leukemic relapses in donor equipped with activating KIRs in haplo-HSCT has been described. Here, we show that KIR3DS1 mediates positive signals upon recognition of HLA-B*51 (Bw4-I80) surface molecules on target cells and that this activation occurs only in Bw4-I80^neg^ individuals, including those carrying particular KIR/HLA combination settings. In addition, killing of HLA-B*51 transfected target cells mediated by KIR3DS1^+^/NKG2A^+^ natural killer (NK) cell clones from Bw4-I80^neg^ donors could be partially inhibited by antibody-mediated masking of KIR3DS1. Interestingly, KIR3DS1-mediated recognition of HLA-B*51 could be better appreciated under experimental conditions in which the function of NKG2D was reduced by mAb-mediated blocking. This experimental approach may mimic the compromised function of NKG2D occurring in certain viral infections. We also show that, in KIR3DS1^+^/NKG2A^+^ NK cell clones derived from an HLA-B Bw4-T80 donor carrying 2 *KIR3DS1* gene copy numbers, the positive signal generated by the engagement of KIR3DS1 by HLA-B*51 resulted in a more efficient killing of HLA-B*51-transfected target cells. Moreover, in these clones, a direct correlation between KIR3DS1 and NKG2D surface density was detected, while the expression of NKp46 was inversely correlated with that of KIR3DS1. Finally, we analyzed KIR3DS1^+^/NKG2A^+^ NK cell clones from a HLA-B Bw4^neg^ donor carrying cytoplasmic KIR3DL1. Although these clones expressed lower levels of surface KIR3DS1, they displayed responses comparable to those of NK cell clones derived from HLA-B Bw4^neg^ donors that expressed surface KIR3DL1. Altogether these data suggest that, in particular KIR/HLA combinations, KIR3DS1 may play a role in the process of human NK cell education.

## Introduction

Natural killer (NK) cells are lymphocytes of innate immunity that are involved in the host immune defenses against viruses and tumor cells. NK cells can exert cytotoxicity against transformed cells and release soluble factors important for regulating innate and adaptive immune responses (1, 2).

The NK cell function is controlled by an array of activating and inhibitory receptors, including the family of killer-cell immunoglobulin-like receptors (KIRs) (3). Inhibitory KIRs (iKIRs) possess a long cytoplasmic tail, containing ITIM motifs, responsible for the transduction of an inhibitory signal (3, 4), while activating KIRs (aKIRs) are characterized by a short tail and by a positively charged amino acid residue in their transmembrane region, which allows recruitment of the DAP12 signaling adaptor molecule (5, 6).

*KIR3DL1/S1* is the *KIR* gene characterized by the highest degree of polymorphism, and it is the only one including alleles coding for either inhibitory (KIR3DL1) or activating (KIR3DS1) receptors. In particular, KIR3DL1 is highly polymorphic, whereas KIR3DS1*013 is the most represented in all examined populations (7). Notably, KIR3DS1 is the only activating receptor with three extracellular domains (8). Its inhibitory counterpart, KIR3DL1, recognizes HLA-A and HLA-B alleles, sharing the Bw4 public epitope (3, 4, 9, 10). Despite the high degree of homology between these two KIR3D receptors, knowledge about KIR3DS1 function and ligand specificity is not completely defined so far.

In this regard, several studies have suggested that certain HLA-B Bw4 alleles characterized by isoleucine in position 80 (Bw4-I80) may be putative ligands for KIR3DS1. In particular, the carriage of a *KIR3DS1* allele in conjunction with *HLA-Bw4-I80* alleles in patients with chronic HIV-1 infection has been associated with a slower progression to AIDS (11, 12). In addition, in individuals affected by acute HIV-1 infections and carrying *HLA-Bw4-I80* alleles, expansion of KIR3DS1^+^ NK cells (13), killing of HIV-1 infected cells, and inhibition of viral replication have been reported (12).

Carr and colleagues (14) could not detect any KIR3DS1-Fc binding to LCL721.221 cells transfected with HLA-B Bw4-I80 alleles (HLA-B*57:01, HLA-B*58:01) or with HLA-B Bw4-T80 (B*27:05) and HLA-Bw6 (B*15:02). Nevertheless they did not exclude the possibility that binding occurred below their detection limits or required the presence of additional factors, such as the presence of specific peptides in the HLA-B peptide-binding groove. In this regard, it has been shown that KIR3DS1 can interact productively with HLA-Bw4 in the context of HIV infection. Indeed, two HIV-derived peptides have been described to enable HLA-B*57:01/KIR3DS1 interaction (15).

Recent studies have also reported that HLA-F open conformers (OCs) are high-affinity ligands of KIR3DS1 and ligands of lower affinity for the inhibitory receptors KIR3DL1 and KIR3DL2 (16, 17). However, KIR3DS1/HLA-F interaction cannot fully explain the control of HIV infection in KIR3DS1^+^ HLA-B Bw4-I80^+^ patients only (16).

A protective role of KIR3DS1 in controlling certain tumors promoted by chronic viral infections has also been observed. For example, a protective effect of *KIR3DS1* in combination with *HLA-B Bw4-I80* alleles has been observed against hepatocellular carcinomas developed in chronically HCV-infected patients (18).

Remarkably, despite several attempts to define the specificity of KIR3DS1, the role of this receptor in the process of NK cell education has not been considered yet. In the present study, by the analysis of distinct NK clones, we show that certain self HLA-B allotypes can modify the functional responsiveness of KIR3DS1, thus providing evidence for an effect on the education of NK cells expressing this aKIR.

## Materials and Methods

### *KIR* Gene Profile and *KIR*-Ligand (*KIR*-L) Analyses

DNA of the tested donors was extracted using QIAamp DNA Blood Mini Kit (Qiagen, GmbH, Germany). The *KIR* gene profile and *KIR*-L analyses were performed using sequence-specific primer PCR (SSP-PCR) KIR genotyping kit and KIR ligand kit, respectively (GenoVision, Saltsjöbaden, Sweden) following the manufacture’s instruction. SSP-PCR analysis of *KIR* gene repertoire has been integrated with sequence of *KIR3DL1* codon 86 in order to distinguish *KIR3DL1* alleles coding for surface receptors from those coding for polypeptide retained into the cytoplasm (19).

### *KIR3DL1* and *KIR3DS1* Gene Copy Number (GCN)

*KIR3DL1* and *KIR3DS1* GCN was measured using a quantitative PCR method and a comparative *C*_t_ method (ΔΔ*C*_t_). The used amplification protocol, as well as primer and probe sequences, has been published by Jiang and coworkers (20). *RNaseP* was used as two copies reference gene (TaqMan Copy Number Reference Assay, human RNase P; Applied Biosystems).

### *HLA-B* High-Resolution Typing

Genomic DNA was used to perform *HLA-B* high-resolution typing. Some *HLA* typings were performed by sequence-based typing (SBT) using ATRIA kits according to the manufacturer’s instructions (Abbott–Celera Corporation, Alameda, CA, USA). Exons 2, 3, and 4 were bidirectionally sequenced using an ABI 3130xl Genetic Analyzer (Applied Biosystems, Foster City, CA, USA), and the sequences were analyzed by Assign 3.5+ HARPS software (Conexio Genomics, Applecross, Australia). Some *HLA* typings were performed using sequence-specific oligonucleotide probes (PCR-SSOP; One Lambda, Canoga Park, CA, USA), and the results were analyzed by the software Fusion 3.0 (One Lambda, Canoga Park, CA, USA).

### Antibodies and Flow Cytometry

The following mAbs, all produced in our lab, were used in this study: c218 (IgG1, anti-CD56), c127 (IgG1, anti-CD16), AZ20 and F252 (IgG1 and IgM, respectively, anti-NKp30), BAB281 and KL247 (IgG1 and IgM, respectively, anti-NKp46), Z231 (IgG1, anti-NKp44), ON72 and BAT221 (IgG1, anti-NKG2D), KRA236 and F5 (IgG1 and IgM, respectively, anti-DNAM-1), 11PB6 (IgG1, anti-KIR2DL1/S1), GL183 (IgG1, anti-KIR2DL2/L3/S2), ECM41 (IgM, anti-KIR2DL3), DF200 (IgG1, anti-KIR2DL1/L2/L3/S1/S2/S5), FES172 (IgG2a, anti-KIR2DS4), z27 (IgG1, anti-KIR3DL1/S1), AZ158 (IgG2a, anti-KIR3DL1/L2/S1), Q66 (IgM, anti-KIR3DL2), z199 and Y9 (IgG2b and IgM, respectively, anti-NKG2A), 6A4 and A6/136 (IgG1 and IgM, respectively, anti-HLA-class I), D1/12 (IgG2a, anti-HLA-DR), 5A10 (IgG1, anti-PVR), L14 (IgG2a, anti-Nectin-2), and BAM195 (IgG1, anti-MICA). F278 (IgG1, anti-LIR-1/ILT2) mAb was kindly provided by Dr. Daniela Pende. Anti-NKG2C (IgG2b, 134522 clone), anti-ULBP-1 (IgG2a, 170818 clone), anti-ULBP-2 (IgG2a, 165903 clone), anti-ULBP-3 (IgG2a, 166510 clone), and anti-KIR2DL1-PE, -FITC, -APC, or non-conjugated (IgG1, 143211 clone) mAbs were purchased from R&D System Inc. (Abingdon, UK). Anti-KIR2DL5-PE or non-conjugated (UP-R1 clone), anti-KIR3DL1-FITC and -APC (DX9 clone) mAbs were purchased from Miltenyi Biotec (Bergisch Gladbach, Germany). Anti-CD3-FITC (UCHT-1 clone), anti-CD56-PC7 (N901 clone), anti-NKG2A-APC (z199 clone), IgG1-PE (679.1Mc7 clone, isotype control), anti-KIR3DL1/S1-PE (z27 clone), and anti-KIR2DL2/L3/S2-PC7 (GL183 clone) mAbs were purchased from Beckman Coulter Immunotech (Marseille, France). Anti-KIR2DL2/L3-S2-FITC (CHL-clone) mAb was obtained from BD Bioscience Pharmingen (San Diego, CA, USA). Anti-HLA-Bw6-FITC and anti-HLA-Bw4-FITC mAbs were purchased from One Lambda (Canoga Park, CA, USA). Anti-human HLA-E (IgG1, 3D12 clone) and anti-human HLA-G (IgG1, MEM-G/9 clone) mAbs were purchased from BioLegend (San Diego, CA, USA) and Abnova (Taipei, Taiwan), respectively.

For cytofluorimetric analyses, cells were incubated with appropriate mAbs, followed by PE-, FITC-, or APC-conjugated isotype-specific goat anti-mouse secondary reagents (Southern Biotechnology Associated, Birmingham, AL, USA; Jackson ImmunoResearch Laboratories, Suffolk, UK) and/or fluorochrome-conjugated mAbs. Cytofluorimetric analyses were performed on FACSCalibur (Becton Dickinson & Co., Mountain View, CA, USA), and data were analyzed by the CellQuest Pro software.

Mean fluorescence intensity ratios (MFIRs) were calculated by dividing the mean fluorescence intensity of stained molecules by the mean fluorescence of the respective isotype control.

### Generation of Resting NK Cells and NK Cell Clones

Buffy coats from healthy donors were obtained from the Immunohematology and Transfusion Center at the S. Martino Hospital (Genova, Italy). Approval was obtained by the ethical committee of IRCCS S. Martino-IST (39/2012) of Genova (Italy). Informed consent was provided according to the Declaration of Helsinki. Human peripheral blood mononuclear cells (PBMCs) were isolated by Ficoll/Hypaque gradients. PBMCs from 120 healthy donors were screened for their KIR3DS1 expression by cytofluorimetric analysis. Donors characterized by the expression of a clearly detectable KIR3DS1^+^ NK cell subset (i.e., Z27^+^ DX9^neg^) were further typed for their KIR repertoires and KIR-Ls (*n* = 40 donors). Based on KIR-L analysis, three groups of donors were selected: Bw4-I80 donors (*n* = 12), Bw4-T80 donors (*n* = 7), and Bw4^neg^ donors (*n* = 8). Individuals carrying both HLA-B Bw4-I80- and Bw4-T80-coding alleles were not considered in this study.

Highly purified NK cells (97–99% purity) were isolated by depletion of non-NK cells, using Miltenyi NK Cell Isolation Kit (Miltenyi Biotec, Bergisch Gladbach, Germany) from some of the selected KIR3DS1^+^ donors (two Bw4-I80, two Bw4-T80, and three Bw4^neg^ donors). NK cells were cultured on irradiated feeder cells in the presence of 100 U/ml rhIL-2 (Proleukin; Chiron Corp., Emeryville, CA, USA) and 2 µg/ml phytoemagglutinin (PHA; Life Technologies, Paisley, UK) in round-bottomed 96-well microtiter plates to obtain activated polyclonal NK cell populations or, after limiting dilution, NK cell clones as previously described (21). After 2–4 weeks of culture, the expanded NK cells were used for the phenotypic analysis and NK cell cytotoxicity experiments. The relatively limited number of donors used to generate NK cell clones reflects the difficult collection of individuals with similar characteristics in terms of KIR and KIR-Ls. Only KIR3DS1^+^ NK cell clones expressing the inhibitory receptor NKG2A as HLA-specific receptor and characterized by a sufficient growth to perform phenotypic and functional tests were selected and analyzed in the study. The number of KIR3DS1^+^/NKG2A^+^ NK cell clones used is indicated in the legends to the figures.

### Analysis of *HLA-F* Transcript

Total RNA was extracted from LCL721.221, C1R, C1R-B51, and JA3 cell lines using RNeasy mini kit (Qiagen) according to the manufacturer’s instruction, and cDNA synthesis was performed using oligo-dT primers. *HLA-F* transcript analysis was performed using Hs04193807_g1: HLA-F human kit (Applied Biosystems, Foster City, CA, USA). *GAPDH* transcript was used to normalize the *HLA-F* quantity (Human GAPDH Endogenous Control Kit, Applied Biosystems, Foster City, CA, USA). The normalized *HLA-F* mRNA transcript of the tested samples was calculated as time-fold mRNA detected in the LCL721.221 cell line (chosen as reference in this study). Each cell line was analyzed in four independent experiments, and each reaction was performed at least in triplicate.

### ^51^Cr Cytolytic Assays

The NK-mediated cytotoxicity was assessed in a 4-h ^51^Cr-release assay as previously described (22). Cells used as targets in the various cytolytic assays were the following: P815 (murine mastocytoma cell line), C1R (human EBV-transformed lymphoblastoid cell line), and C1R transfected with HLA-B*51 (Bw4-I80) allele (Figure S1 in Supplementary Material). For redirected killing assays, P815 were used as target cells in the presence of mAbs of IgG isotype at a concentration of 0.5 µg/mL. For masking experiments, NK cells were pre-incubated with mAbs specific to the various NK receptors 10 min before addition of target cells; mAb concentration was 10 µg/mL. The E:T ratios are indicated in the figure legends. Δ and Δ-B51 indicate the variations of C1R or C1R-B51 lysis in the absence or presence of anti-KIR3DS1 mAb calculated for each NK cell clone.

Three different types of NK cell clones were used as effector cells for cytolytic assays: (1) NKG2A^+^, KIR3DS1^+^, KIR2DL2/S2/L3^neg^, KIR2DL1/S1^neg^, KIR3DL1^neg^, and NKG2C^neg^; (2) KIR3DL1^+^, NKG2A^+^, KIR3DS1^neg^, KIR2DL2/S2/L3^neg^, KIR2DL1/S1^neg^, and NKG2C^neg^; and (3) KIR3DL1^neg^, NKG2A^+^, KIR3DS1^neg^, KIR2DL2/S2/L3^neg^, KIR2DL1/S1^neg^, and NKG2C^neg^.

### Statistical Analysis

Wilcoxon–Mann–Whitney non-parametric tests were employed. The statistical significances (*p* value: *p < 0.1, **p < 0.01, ***p < 0.001) are indicated. Graphic representations and statistical analysis were performed using GraphPad Prism 6 (GraphPad Software, La Jolla, CA, USA).

## Results

### KIR3DS1 Surface Expression on Resting NK Cells Is Not Affected by Differences in HLA-B Allotypes

**Figure 1:**
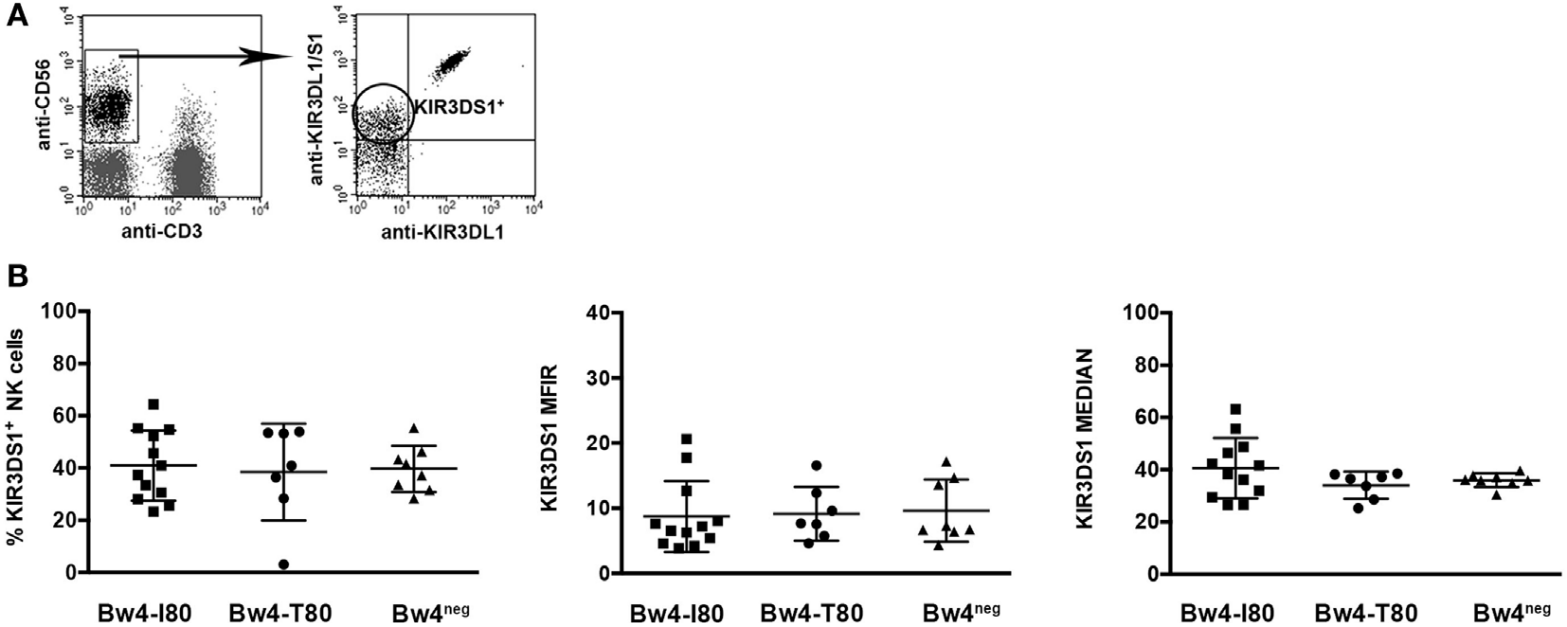
**KIR3DS1 surface expression on resting peripheral blood natural killer (NK) cells**. **(A)** Gate strategy for selecting KIR3DS1^+^ NK cell subset (CD3^neg^ CD56^+^ KIR3DS1^+^ KIR3DL1^neg^) in peripheral blood mononuclear cell. For staining, the following mAbs were used in combination: anti-CD56 (N901), anti-CD3 (UCHT-1), anti-KIR3DL1/S1 (z27), and anti-KIR3DL1 (DX9). **(B)** KIR3DS1 surface expression was evaluated on peripheral blood resting NK cells from HLA-B Bw4-I80 (*n* = 12, full squares), Bw4-T80 (*n* = 7, full circles), or Bw4^neg^ (*n* = 8, full triangles) healthy donors in terms of frequency, mean fluorescence intensity ratio, and median values. Summarized results are compared in three scatter dot plots, respectively. In each plot, average and standard deviation are represented.

Healthy donors were screened for their KIR3DS1 expression by cytofluorimetric analysis. To distinguish between KIR3DS1^+^ and KIR3DL1^+^ cells, peripheral blood NK cells were stained in double fluorescence analysis with two different mAbs: an anti-KIR3DL1-specific mAb (clone DX9) and an mAb specific for both KIR3DL1 and KIR3DS1 (clone Z27) (Figure 1A). Donors characterized by the expression of a KIR3DS1^+^ NK cell subset (i.e., Z27^+^ DX9^neg^) were further typed for their *KIR* repertoires and *KIR*-Ls (Figure S2 in Supplementary Material).

*KIR* gene analysis allowed us to define the telomeric regions of the tested donors. In order to compare donors characterized by similar *KIR3DL1/3DS1* locus, we restricted our analysis to TelA/TelB donors, namely, individuals characterized by one *KIR3DL1* and one *KIR3DS1* gene copy. *KIR3DL1^neg^* donors (i.e., individuals characterized by two B telomeric regions) were excluded from this study according to recent evidences showing hyporesponsive and less frequent KIR3DS1^+^ NK cells in this type of donors (23). Subsequently, based on KIR-L analysis, three groups of donors were selected: (a) Bw4-I80 donors, typed as Bw4-I80^pos^ and Bw4-T80^neg^ (including both Bw4-I80/Bw4-I80 and Bw4-I80/Bw6 donors); (b) Bw4-T80 donors, typed as Bw4-T80^pos^ and Bw4-I80^neg^ (including both Bw4-T80/Bw4-T80 and Bw4-T80/Bw6 donors); and (c) Bw4^neg^ donors, lacking both Bw4-I80 and Bw4-T80. Individuals carrying both HLA-B Bw4-I80- and Bw4-T80-coding alleles were not considered in this study. Also some HLA-A molecules are characterized by a Bw4-80I motif (e.g., HLA-A*23, -A*24, -A*25 or -A*32) (24), and their frequency in the European population is ~16% (http://www.ncbi.nlm.nih.gov/gv/mhc/ihwg.cgi?ID=9&cmd=PRJOV). Nevertheless, since not all HLA-A Bw4-I80 molecules are KIR3DL1 ligands (10, 25), we restricted our analyses to HLA-B alleles with Bw4-I80.

Comparison of KIR3DS1 surface expression on resting NK cells derived from these three donor groups did not show any significant difference in terms of percentage, MFIR, and median values (Figure 1B). These data are in line with previous results showing no substantial differences in *KIR3DS1* mRNA levels between HLA-B Bw4-I80 and HLA-Bw6 healthy individuals (13).

Thus, similar to what had already been demonstrated for HLA-C alleles in relation to KIR2DS1 (26), expression of different HLA-B allotypes does not influence the overall frequency of KIR3DS1^+^ NK cells and the KIR3DS1 surface expression density in peripheral blood NK cells.

### Phenotypic and Functional Differences in KIR3DS1^+^/NKG2A^+^ NK Cell Clones Derived from Donors Carrying Various HLA-B Allotypes

**Figure 2:**
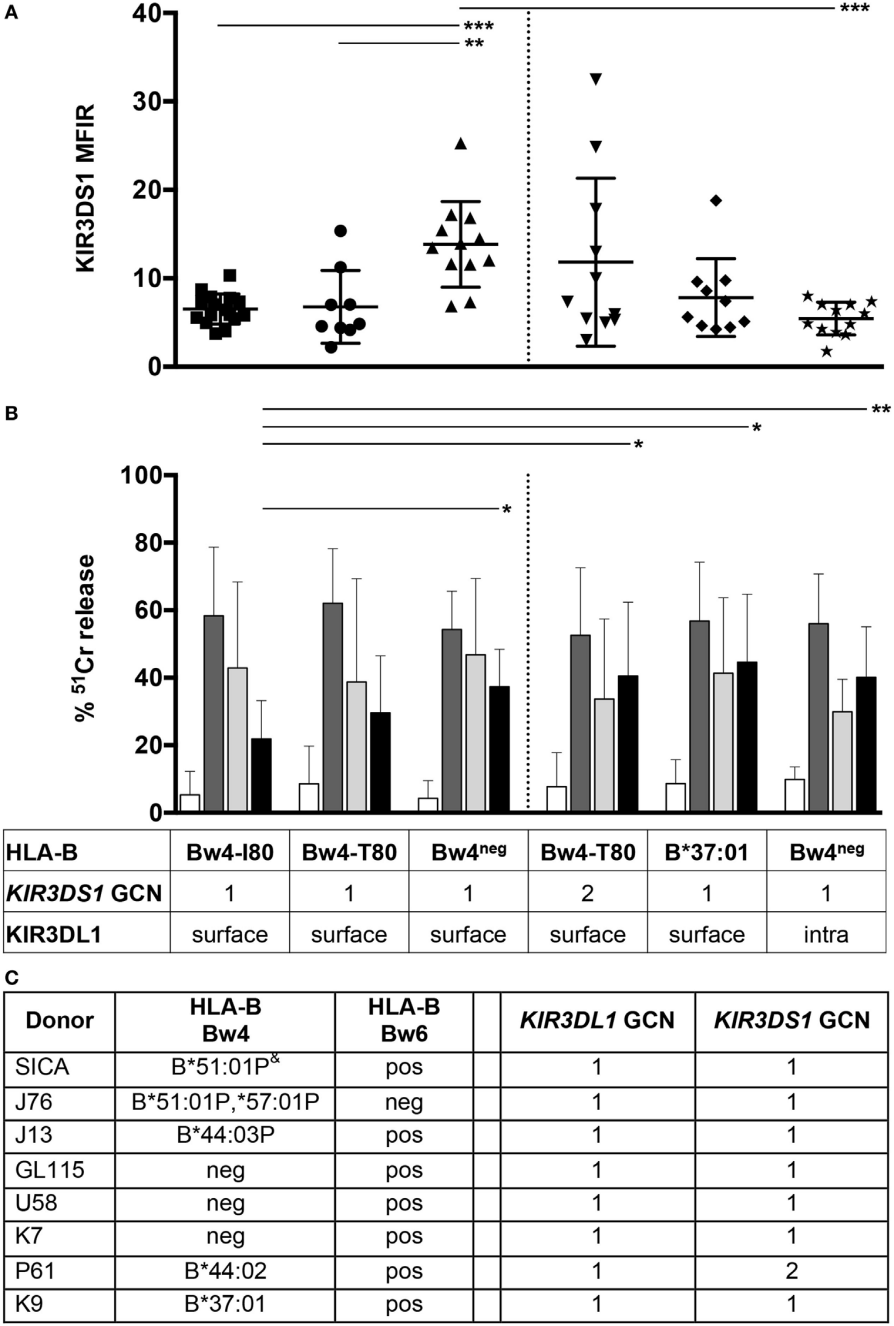
**KIR3DS1 surface expression and mAb-mediated cross-linking of KIR3DS1 in KIR3DS1^+^/NKG2A^+^ natural killer (NK) cell clones**. KIR3DS1^+^/NKG2A^+^ NK cell clones derived from HLA-B-typed donors were analyzed for KIR3SD1 surface expression **(A)** and for ^51^Cr release in redirected killing assay against P815 cell line **(B)**. Data related to HLA typing and surface/intra *KIR3DL1* presence are indicated in the box below panel **(B)**. **(A)** The NK cell clones of the first set (left panel) were derived from Bw4-I80 (*n* = 10, full square), Bw4-T80 (*n* = 9, full circle), and Bw4^neg^ (*n* = 12, full triangles) donors, whereas NK cell clones of the second set (right panel) were derived from T80-3DS1-2GCN (*n* = 11, overturned full triangle), HLA-B*37:01 (*n* = 10, full rhombus), and Bw4^neg^ KIR3DL1^intra^ (*n* = 12, full star) donors. **(B)** The cytolytic activity of the first (left panel) and second (right panel) set of KIR3DS1^+^/NKG2A^+^ NK cell clones was evaluated in redirected killing assays upon mAb-mediated cross-linking of different NK receptors. Cytolytic assays were performed in the absence of mAbs (white bar) or in the presence of anti-CD16 (c127, dark gray bar), anti-NKp46 (BAB281, light gray bar), or anti-KIR3DS1 (z27, black bar) mAbs. Average, standard deviation, and *p* values are indicated (*p < 0.1, **p < 0.01, and ***p < 0.001). **(C)** HLA-B typing, *KIR3DL1*, and *KIR3DS1* gene copy number (GCN) analyses of the indicated donors are shown.^&^P: indicated a group of alleles with identical sequence at exons 2 and 3 and therefore sharing the same antigen-binding domains.

Several NK cell clones were generated from donors belonging to each of the three above-mentioned groups (Bw4-I80: SiCa and J76 donors, Bw4-T80: J13 donor, and Bw4^neg^: GL115 and U58 donors). These donors were all characterized by a TelA/TelB *KIR* genotype, nevertheless, since *KIR* haplotype characterized by *KIR3DL1/3DS1* duplication has been described (20, 27), GCN analysis was performed to verify, in these five donors, the presence of one *KIR3DL1* and *KIR3DS1* copy (Figure 2). To exclude the potential educational effects of different KIR/KIR-L interactions on KIR3DS1 expression and function, KIR3DS1^+^ NK cell clones expressing the inhibitory receptor NKG2A as the only known HLA-specific receptor were selected.

As shown in Figure 2A (left panel), cytofluorimetric analysis revealed that KIR3DS1 expression was significantly heterogeneous among NK cell clones derived from individuals with different HLA-B allotypes. Indeed, NK cell clones derived from Bw4-I80 donors, and to a lower extent those from Bw4-T80 donors, showed a reduced expression of KIR3DS1 as compared to NK cell clones derived from Bw4^neg^ donors (***p < 0.0001 and **p = 0.0018, respectively). On the contrary, no differences in NKG2A surface expression could be detected among the same series of NK cell clones analyzed (not shown).

**Figure 3:**
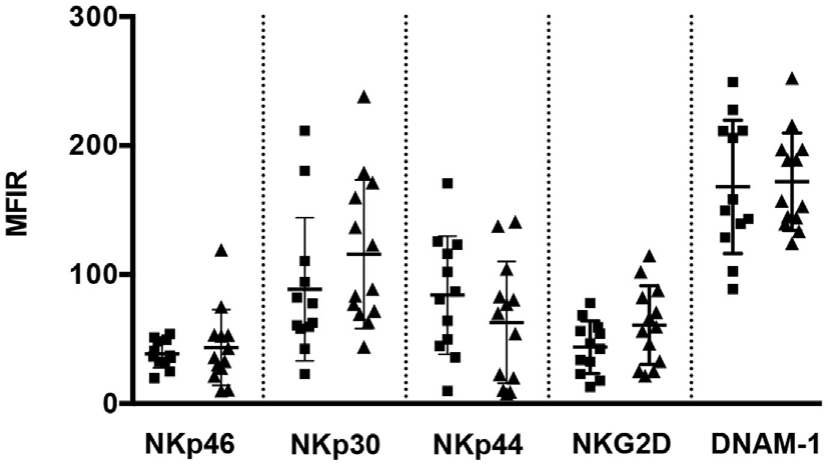
**Surface expression of several non-HLA-specific activating receptors**. Staining of NKp46 (BAB281), NKp30 (AZ20), NKp44 (z231), NKG2D (ON72), and DNAM-1 (KRA236) molecules on KIR3DS1^+^/NKG2A^+^ natural killer (NK) cell clones derived from Bw4-I80 (*n* = 12, full squares) or Bw4^neg^ carrying surface KIR3DL1 (*n* = 13, full triangles) donors were compared in terms of mean fluorescence intensity ratio (MFIR) in scatter dot plot representation. Average and standard deviation are shown.

KIR3DS1^+^/NKG2A^+^ NK cell clones were then analyzed in redirected killing assays using mAbs specific for several activating or inhibitory receptors. Notably, the magnitude of cytolytic responses to KIR3DS1 mAb-mediated triggering correlated with the levels of KIR3DS1 surface expression. In particular, as shown in Figure 2B (left panel), NK cell clones from Bw4-I80 donors expressing a significantly lower KIR3DS1 MFIR displayed a reduced increment of cytotoxicity upon KIR3DS1 mAb-mediated cross-linking as compared to those derived from Bw4^neg^ donors (*p = 0.0287). On the contrary, mAb-mediated triggering of other activating receptors, including NKp46, CD16 (Figure 2B, left panel), NKp30, NKp44, NKG2D, and DNAM-1 (not shown), did not display any significant variation between KIR3DS1^+^ NK cell clones derived from Bw4-I80 and those derived from Bw4^neg^ donors. Notably, the expression of these (non-HLA-specific) activating receptors did not display any significant difference in KIR3DS1^+^ NK cell clones derived from Bw4-I80 or Bw4^neg^ donors (Figure 3).

### Phenotypic and Functional Analysis of KIR3DS1^+^/NKG2A^+^ NK Cell Clones from Donors with Peculiar *KIR3DL1/S1* and *HLA-B* Combinations

Considering that it has been shown that the number of *KIR3DL1* and *KIR3DS1* gene copies plays an important role in modulating the HIV-1 control and that this effect seems to be detectable only after epistatic interactions between HLA molecules and KIRs (23, 28), we extended the phenotypic and functional analyses to additional NK cell clones derived from donors with peculiar *KIR3DL1/S1* and *HLA-B* combinations [Figures 2A,B (right panels) and Figure 2C]. In particular, NK cell clones were derived from: (a) an HLA-B Bw4-T80/Bw6 donor equipped with 2 *KIR3DS1* and 1 *KIR3DL1* GCN (P61, referred to as T80-3DS1-2GCN); (b) an HLA-B Bw4-T80/Bw6 donor carrying HLA-B*37:01, a particular Bw4-T80 allotype characterized by D77-T80 sequence (K9, referred to as HLA-B*37:01); and (c) an HLA-Bw4^neg^ donor equipped with a *KIR3DL1* allele coding for a polypeptide retained into the cytoplasm (K7, referred to as Bw4^neg^ KIR3DL1^intra^) (29). As shown in Figure 2A (right panel), comparison of KIR3DS1 MFIR among NK cell clones of this second set (T80-3DS1-2GCN, HLA-B*37:01, Bw4^neg^ KIR3DL1^intra^ donors) did not reveal significant differences. On the contrary, some differences could be detected by comparing the first and the second set of donors analyzed. As shown in Figure 2A, KIR3DS1^+^ NK cell clones derived from T80-3DS1-2GCN donor displayed a more heterogeneous KIR3DS1 surface expression as compared to NK cell clones derived from T80 donor carrying one *KIR3DS1* GCN. Nevertheless, NK cell clones derived from T80-3DS1-2GCN donor (but also those from B*37:01 donor) did not display significant differences in terms of KIR3DS1 MFIR as compared to NK clones of the first set.

On the contrary, NK cell clones from the Bw4^neg^ KIR3DL1^intra^ donor displayed a significantly lower KIR3DS1 surface expression (***p < 0.0001) (Figure 2A) but a similar cytotoxicity upon KIR3DS1 mAb-mediated triggering (Figure 2B) as compared to NK cell clones derived from Bw4^neg^ donors expressing surface KIR3DL1.

Moreover, the comparison of the cytotoxicity in redirected killing assays between the first and second sets of donors revealed that NK cell clones derived from the second set were characterized by higher increments of cytotoxicity upon KIR3DS1 mAb-mediated triggering than NK cell clones from Bw4-I80 donors (*p = 0.0243 with T80-3DS1-2GCN donor, *p = 0.0014 with HLA-B*37:01 donor, and **p = 0.0059 with Bw4^neg^ KIR3DL1^intra^ donor) (Figure 2B).

### Correlation Analysis between Expression of KIR3DS1 and Non-HLA-Specific Activating Receptors in Donors Characterized by Different HLA-B Allotypes

**Figure 4:**
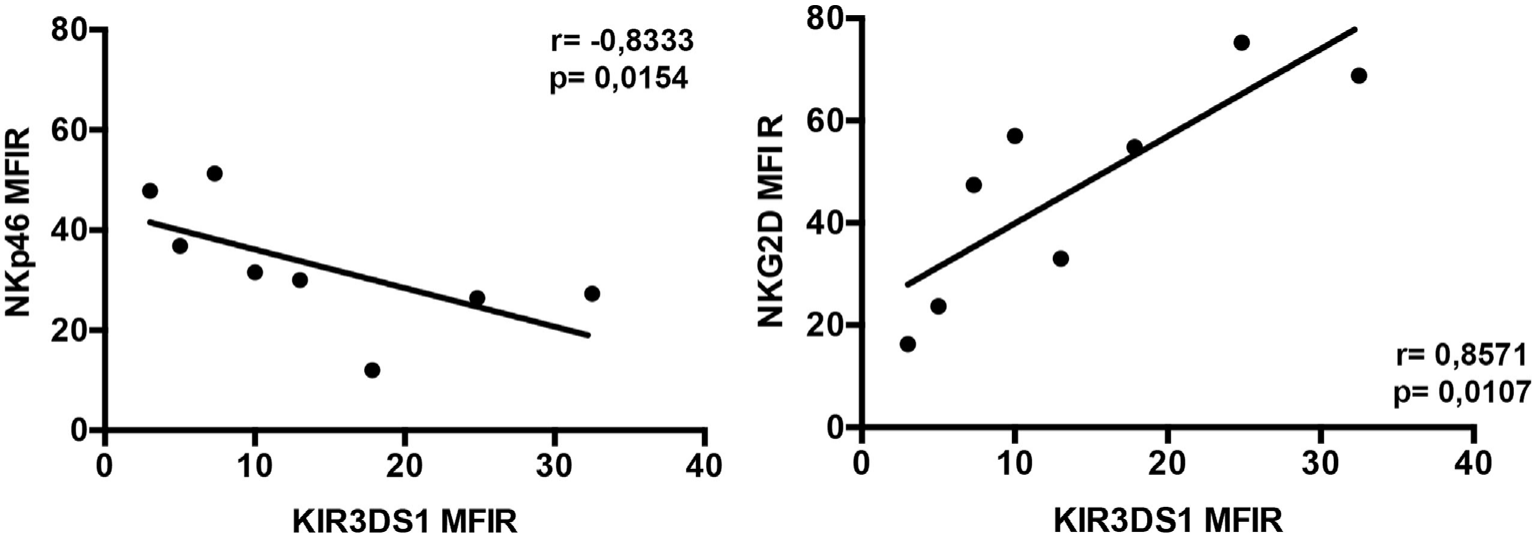
**Phenotypic analyses of KIR3DS1^+^/NKG2A^+^ natural killer (NK) cell clones derived from T80-3DS1-2GCN donor**. Correlation analyses among NKp46 or NKG2D activating receptors and KIR3DS1 surface expression in several KIR3DS1^+^/NKG2A^+^ NK cell clones (*n* = 8) derived from a T80-3DS1-2GCN donor are represented. Linear regression values (*r*) and *p* values are shown.

The existence of a possible correlation between the expression of KIR3DS1 and that of relevant non-HLA-specific activating receptors (i.e., NCRs, NKG2D and DNAM-1) was analyzed in all KIR3DS1^+^/NKG2A^+^ NK cell clones considered in the present study. Of interest, only in T80-3DS1-2GCN donor, KIR3DS1 surface density correlated inversely with NKp46 and directly with NKG2D MFIR (*p = 0.0154 and *p = 0.0107, respectively) (Figure 4). Notably, this result was detected only in NK cell clones from these donors characterized by higher and heterogeneous values of KIR3DS1 MFIR (Figure 2A, right panel). Based on this observation, it cannot be ruled out that, in given HLA/KIR haplotype settings, KIR3DS1 may influence the surface density of NKp46 and NKG2D.

### KIR3DS1-Mediated Recognition of HLA-B*51 Allele on Transfected Cells

According to previous data suggesting a possible interaction between KIR3DS1 and HLA-B Bw4-I80 alleles (30) and considering the high level of homology between the extracellular domains of KIR3DS1 and KIR3DL1 as well as the ability of KIR3DL1 to recognize in most instances HLA-B Bw4-I80 alleles with higher efficiency than HLA-B Bw4-T80 alleles (9, 25), all KIR3DS1^+^/NKG2A^+^ NK cell clones were assessed for their capability of killing HLA-B Bw4-I80 target cells. In particular, the killing activity of KIR3DS1^+^/NKG2A^+^ NK cell clones was assessed against the C1R cell line transfected or not with HLA-B*51 allele (31).

**Figure 5:**
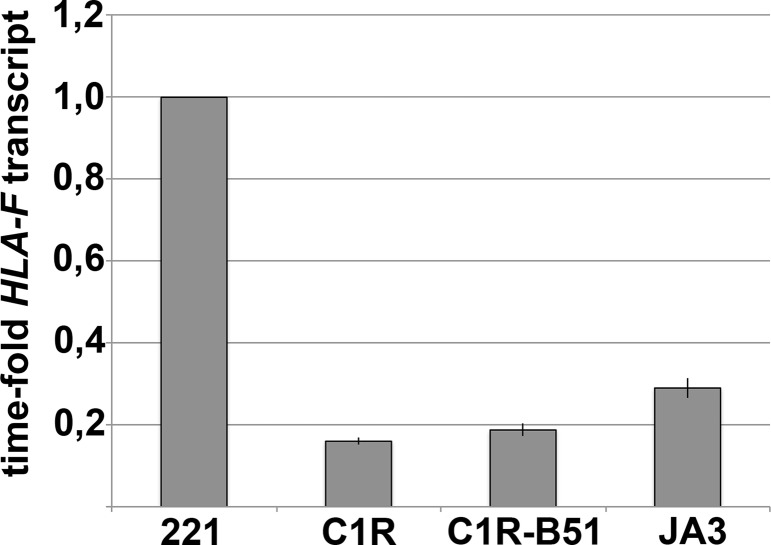
**C1R and C1R-B51 express low level of *HLA-F* transcript**. *HLA-F* mRNA amount detected in the analyzed cell lines has been normalized to *GAPDH* transcript. The normalized *HLA-F m*RNA transcript of the tested cell lines was calculated as time-fold the mRNA detected in LCL721.221 cell line (chosen as reference). Mean values obtained in four different experiments and their standard deviations are reported.

Considering recent studies showing that HLA-F OCs are high-affinity ligands of KIR3DS1 (16, 17), the *HLA-F* transcript was analyzed in C1R and C1R-B51 target cells to assess whether these cells would express this KIR3DS1 ligand. JA3 (a Jurkat clone) (32) and LCL 721.221 cell lines were used as negative control and positive control, respectively (16, 33). As shown in Figure 5 and Figure S3 in Supplementary Material, C1R and C1R-B51 expressed very low levels of *HLA-F* transcript similar to JA3 cells, whereas LCL 721.221 expressed *HLA-F* mRNA five times more than C1R-B51. Thus, possible differences of lysis between C1R and C1R-B51 may not be attributed to recognition of HLA-F by KIR3DS1.

**Figure 6:**
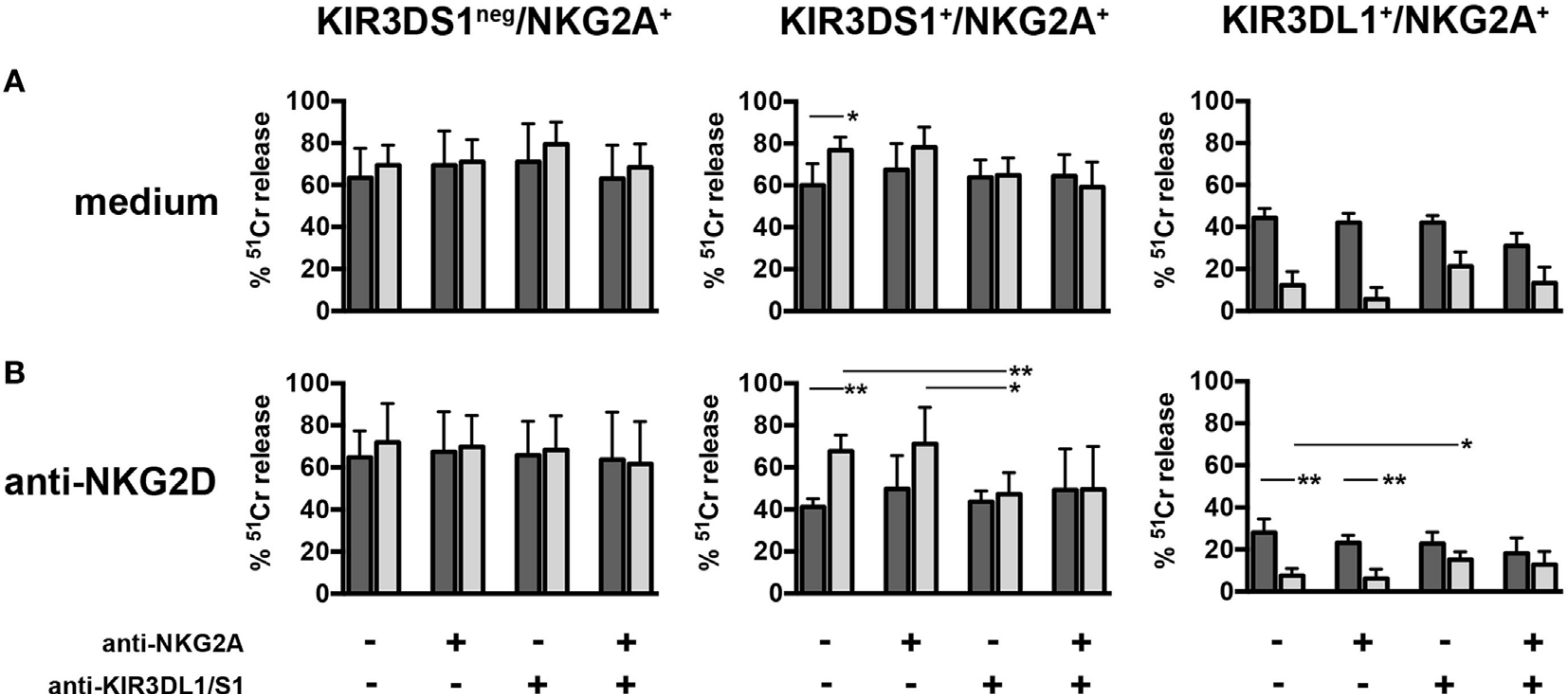
**Killing of C1R-B51 target cells by natural killer (NK) cell clones derived from a T80-3DS1-2GCN donor**. Three representative NK cell clones (one KIR3DS1^neg^/NKG2A^+^, one KIR3DS1^+^/NKG2A^+^ and one KIR3DL1^+^/NKG2A^+^) derived from the T80-3DS1-2GCN donor were tested for cytotoxic activity against C1R (dark gray histograms) and C1R-B51 (light gray histograms) target cell lines. Experiments were performed in the absence **(A)** or in the presence of anti-NKG2D masking mAb **(B)**. Additional mAb-mediated maskings were carried out as indicated below panel B. Histograms summarized results of three independent experiments in duplicate. Average, standard deviation, and *p* values are shown (*p < 0.1 and **p < 0.01).

A representative cytolytic experiment against C1R/C1R-B51 target cells performed using as effector cell a KIR3DS1^+^/NKG2A^+^ NK cell clone derived from the T80-3DS1-2GCN donor is shown in Figure 6. As controls, KIR3DS1^neg^/NKG2A^+^ and KIR3DL1^+^/NKG2A^+^ NK cell clones derived from the same donor were analyzed. In the absence of mAbs, C1R-B51 cells were killed slightly more efficiently than un-transfected C1R by the KIR3DS1^+^/NKG2A^+^ NK cell clone (*p = 0.0159). Notably, this difference of lysis was abolished upon mAb-mediated masking of KIR3DS1, suggesting a possible positive recognition of HLA-B*51 by KIR3DS1 (Figure 6A). Interestingly, this result was more evident when the experiment was performed in the presence of anti-NKG2D mAb. Thus, as shown in Figure 6B, the difference between C1R and C1R-B51 killing was amplified by NKG2D mAb-mediated blocking (**p = 0.0022). Importantly, further masking of KIR3DS1 abrogated this difference (**p = 0.0065) (Figure 6B). Similar data were obtained upon additional mAb-mediated masking of NKG2A.

**Figure 7:**
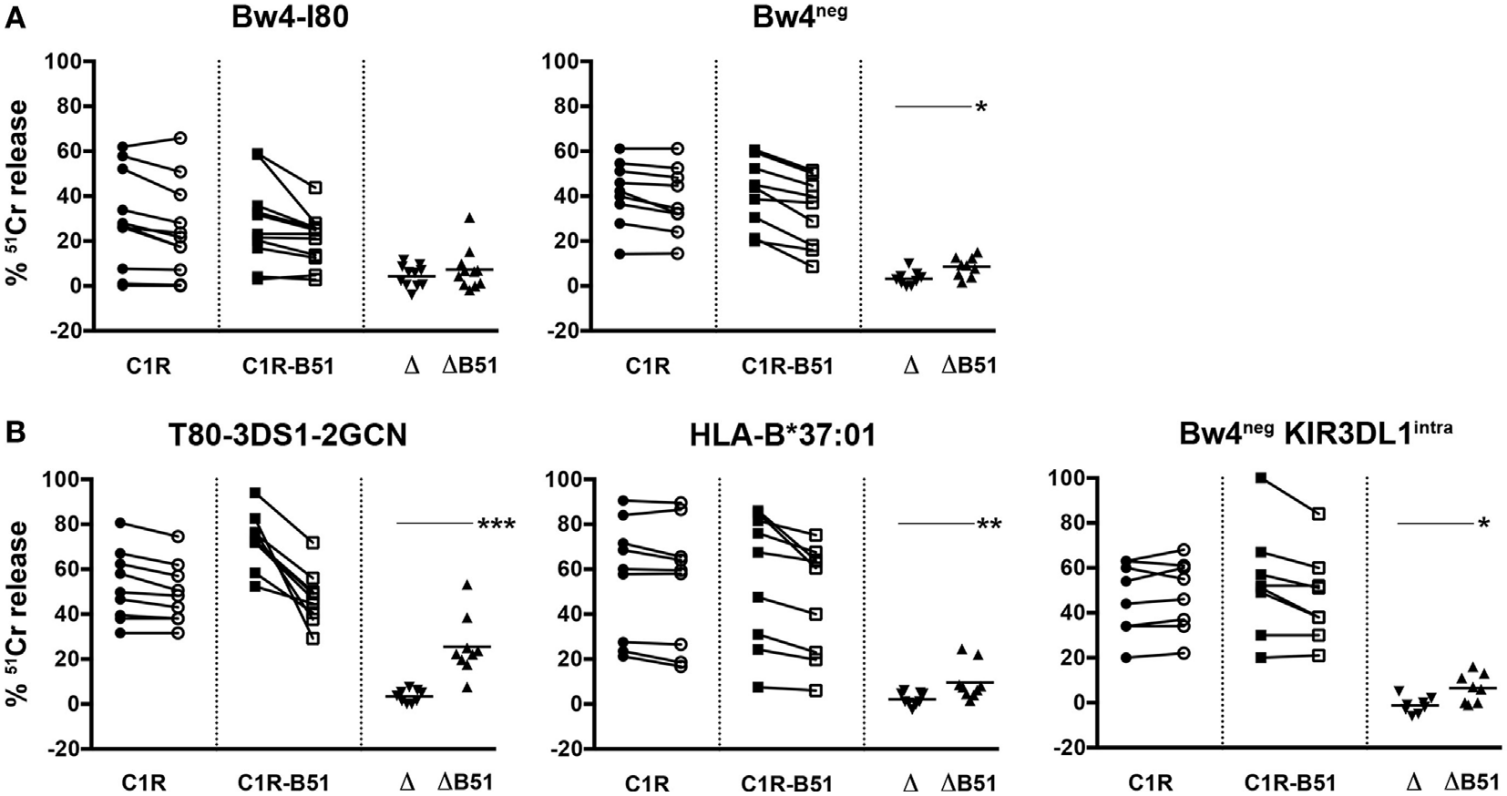
**C1R-B51 killing by KIR3DS1^+^/NKG2A^+^ natural killer (NK) cell clones**. NK cell clones were tested in ^51^Cr-release cytotoxicity assay against C1R (circle) and C1R-B51 (square) target cell lines in the presence of anti-NKG2D + anti-NKG2A masking mAbs (full symbols) or anti-NKG2D + anti-NKG2A + anti-KIR3DS1 masking mAbs (empty symbols). Δ and Δ-B51 indicate the variations of C1R or C1R-B51 lysis in the absence or presence of anti-KIR3DS1 mAb for each NK cell clone. NK cell clones derived from Bw4-I80 (*n* = 11) and Bw4^neg^ (*n* = 9) donors are represented in panel **(A)**, whereas those derived from T80-3DS1-2GCN (*n* = 9), HLA-B*37:01 (*n* = 9), and Bw4^neg^ KIR3DL1^intra^ (*n* = 8) donors are represented in panel **(B)**. Plots summarized results of three independent experiments in duplicate. *p* values indicate a statistically significant difference between the groups (*p < 0.1, **p < 0.01, and ***p < 0.001).

A similar experimental approach was applied to the NK cell clones generated from the other donors analyzed (Figure 7). These clones were characterized by a substantially homogeneous NKG2D expression. The cytolytic assays were performed not only in the presence of blocking NKG2D but also upon mAb-mediated masking of NKG2A in order to further reduce possible functional variations caused by different intensities of inhibitory signals consequent to NKG2A/HLA-E interaction. Then, cytotoxicity was evaluated upon additional blocking of KIR3DS1 (Figure 7). In this set of experiments, in order to better appreciate the KIR3DS1 contribution to target killing, each NK cell clone was also analyzed for possible variations in the lysis of C1R or C1R-B51 targets in the absence or in the presence of KIR3DS1 mAb-mediated blocking (Δ_C1R_ and Δ_C1R-B51_).

In Bw4-I80 donors, lysis of C1R and C1R-B51 was not affected by the addition of anti-KIR3DS1 mAb (as shown by the raw data as well as Δ_C1R_ and Δ_C1R-B51_ values) (Figure 7A). On the other hand, in Bw4^neg^ donors, the addition of anti-KIR3DS1 mAb slightly decreased C1R-B51 killing and a significant gap between Δ_C1R_ and Δ_C1R-B51_ could be detected (*p = 0.0188).

KIR3DS1^+^/NKG2A^+^ NK cell clones from the T80-3DS1-2GCN donor killed more efficiently C1R-B51 than C1R. Moreover, the addition of anti-KIR3DS1 mAb abolished this difference. Remarkably, in this donor, the comparison between Δ_C1R_ and Δ_C1R-B51_ showed a highly significant difference (***p < 0.0001, Figure 7B). Significant differences between Δ_C1R_ and Δ_C1R-B51_ could also be observed in KIR3DS1^+^/NKG2A^+^ NK cell clones derived from both HLA-B B*37:01/Bw6 and Bw4^neg^ KIR3DL1^intra^ donors (**p = 0.0073 and *p = 0.0110, respectively). All together, these data suggest a direct involvement of KIR3DS1 in the recognition of HLA-B*51 (Bw4-I80) target cells. Remarkably, this result can be detected only when NK cells were generated in particular KIR/HLA combination settings. Indeed, KIR3DS1-mediated recognition of HLA-B*51 occurs only when NK cell clones were derived from Bw4-I80^neg^ donors (Bw4-T80-2GCN, B*37:01/Bw6, or Bw4^neg^), suggesting a role for HLA-B/KIR3DS1 interaction in the process of NK cell education.

**Figure 8:**
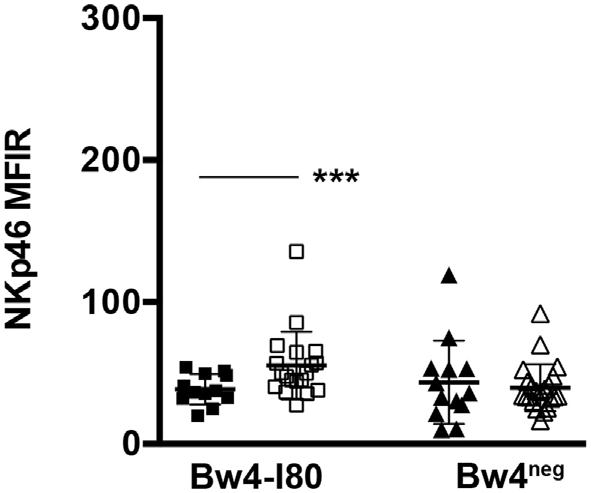
**NKp46 surface expression on KIR3DS1^+^/NKG2A^+^ and KIR3DS1^neg^/NKG2A^+^ natural killer (NK) cell clones derived from Bw4-I80 or Bw4^neg^ donors**. KIR3DS1^+^/NKG2A^+^ (full symbols) and KIR3DS1^neg^/NKG2A^+^ (empty symbols) NK cell clones derived from Bw4-I80 (squares, *n* = 12 and *n* = 19, respectively) and Bw4^neg^ (triangles, *n* = 13 and *n* = 22 respectively) donors were stained with anti-NKp46 mAb (BAB281). Results are summarized in scatter plot analysis. Average, standard deviation, and *p* values are indicated (***p < 0.001).

Notably, when comparing KIR3DS1^+^/NKG2A^+^ and KIR3DS1^neg^/NKG2A^+^ NK cell clones, a significant difference in NKp46 surface expression was observed only in Bw4-I80 donors. Thus, KIR3DS1^+^/NKG2A^+^ NK cell clones expressed lower levels of NKp46 than KIR3DS1^neg^/NKG2A^+^ NK cell clones (***p = 0.0005), further corroborating the possibility that HLA-B/KIR3DS1 interaction may be involved in the process of NK cell education (Figure 8).

## Discussion

A number of studies described an association between given KIR/HLA gene combinations and clinical outcome in various immune challenges and reported a possible perturbation of KIR/HLA interactions by the presented peptide. In particular, an important combined role played by KIR3DS1 and HLA-B Bw4-I80 in controlling HIV infection (11, 12) and the recognition of specific HIV-derived peptides associated with HLA-B*57 alleles by KIR3DS1 have been described (15).

In the present study, we provided the first evidence of a direct involvement of KIR3DS1 in the NK-mediated recognition of HLA-B*51 surface molecules expressed on target cells. This capability is manifested only when KIR3DS1 is expressed by NK cells derived from individuals carrying particular KIR/HLA combinations. Although our study was performed on a limited number of donors (due to the difficulty in the collection of individuals with similar characteristics in terms of KIR and KIR-Ls as well as in the generation and expansion of appropriate NK cell clones), our results suggest a role for this activating receptor in the process of NK cell education. In particular, the KIR3DS1-mediated positive recognition of HLA-B*51 (Bw4-I80) could be detected in NK cell clones derived from Bw4-I80^neg^ donors (one Bw4-T80-2GCN, one B*37:01/Bw6, and three Bw4^neg^) but not in those from Bw4-I80 donors (two donors). Thus, in a manner reminiscent of that previously described for KIR2DS1 (26, 34), the interaction of KIR3DS1 with its self-HLA-B class I ligand (Bw4-I80 alleles) would affect the subsequent response mediated by this aKIR. Indeed, in HLA-B Bw4-I80 donors, the process of NK cell education that is taking place via NKG2A/self-HLA-E interaction is characterized by a decrement in NK cell responsiveness upon KIR3DS1 engagement, thus ensuring self-tolerance. In this context, it has to be considered that KIR3DS1 responsiveness in redirected killing assays was lower in Bw4-I80 than in Bw4-I80^neg^ donors. In particular, among those analyzed, KIR3DS1 was the only receptor showing a significant differential expression and responsiveness between Bw4-I80 and Bw4^neg^ donors.

The analysis performed in the present study was limited to KIR3DS1^+^/NKG2A^+^ NK cell clones in order to evaluate whether KIR3DS1 expression could confer alloreactivity to non-alloreactive NKG2A^+^ NK cells and to minimize the effect that other self KIR/HLA interactions would exert on NK cell education. Several studies have suggested that, in given donor/recipient pairs, the expression of aKIRs, such as KIR2DS1, can amplify the size of the alloreactive NK cell subset. This effect is particularly relevant in the successful therapy of high-risk acute leukemias in the haplo-HSCT setting (35–39). Indeed, it has been shown that the “non-alloreactive” NKG2A^+^ iKIRs^neg^ NK cells can display alloreactivity against HLA-C2^+^ recipient cells when they co-express KIR2DS1 (40). A similar effect could be true, at least in part, also for KIR3DS1. In this context, the definition of the specificity/function of KIR3DS1 would have important implications not only to identify donors capable of generating alloreactive NK cells (on the basis of the existence of a KIR/HLA-class I mismatch) but also to select the best donor, according to the size of the alloreactive NK cell subset.

On the basis of a recent study (23) showing that KIR3DL1-dependent licensing of NK cells could be involved in shaping a strong antiviral response mediated by KIR3DS1^+^ NK cells, we also analyzed KIR3DS1 expression and responsiveness in donors characterized by peculiar KIR/HLA combinations. In this context, we showed that KIR3DS1 mAb-mediated triggering of cytotoxicity in NK cell clones from a Bw4^neg^ donor carrying KIR3DL1^intra^ is similar to that detectable in NK cell clones from Bw4^neg^ donors expressing surface KIR3DL1. It is noteworthy that this increment of cytotoxicity occurred despite the low surface expression of KIR3DS1 (Figure 2). Remarkably, NK cell clones from the Bw4^neg^ donor carrying KIR3DL1^intra^ were able to kill HLA-B*51^+^ target cells with efficacy comparable to that of NK cell clones derived from Bw4^neg^ donors with surface KIR3DL1.

Moreover, different from KIR3DS1^+^ NK cell clones of classical HLA-B Bw4-T80 donors, clones derived from a donor carrying the HLA-B*37 allele (a particular HLA-Bw4-T80 allotype characterized by the D77-T80 sequence) were characterized by higher KIR3DS1 responsiveness in redirected killing assays and higher efficiency of killing HLA-B51^+^ target cells as compared to NK clones from HLA-B Bw4-I80 donors.

However, the highest increment of cytotoxicity against C1R-B51 cells was detected in NK cell clones from a T80-Bw4 donor carrying two copies of *KIR3DS1* and one copy of *KIR3DL1*. Interestingly, in this donor, an inverse correlation between KIR3DS1 and NKp46 MFIR could also be detected. This finding is relevant in the context of NK cell education. Indeed, a low surface expression of NKp46 combined with high KIR3DS1 surface density on NK cell clones may prevent possible KIR3DS1-mediated autoreactive responses in non-pathological conditions. In the same donor, a direct correlation between KIR3DS1 and NKG2D surface expression was also observed. Notably, these correlations could be observed only in NK clones from this donor in which KIR3DS1 surface expression was more heterogeneous and higher as compared to Bw4-T80 donor carrying one copy of *KIR3DS1* and one copy of *KIR3DL1*.

The direct correlation between KIR3DS1 and NKG2D surface expression is particularly interesting if we consider that NKG2D pathway is less functional in certain viral infections. Indeed, previous studies have described that several viral immune evasion strategies possibly evolved to elude NKG2D-mediated immune-surveillance (30, 41–44). For example, the HIV Nef protein prevents the expression of some NKG2D ligands at the surface of infected cells (45, 46). According to these data, in an attempt to simulate the compromised NKG2D function occurring during certain viral infections, we analyzed the effect of mAb-mediated NKG2D blocking in KIR3DS1^+^ NK clones. Thanks to this experimental approach, we could show that KIR3DS1 function may be crucial, primarily during viral infections in which other triggering signals, such as those through NKG2D, are compromised. In this context, it is also important to take into consideration that the expression of HLA-B can be modified only marginally during certain viral infections. Thus, in HIV-1-infected viremic patients, while HLA-A and -Bw6 surface molecules were significantly downmodulated in T cell blasts, HLA-B Bw4 alleles were not (47).

Considering the “discontinuity theory for immunity” (48), it is also possible that KIR3DS1^+^ NK cells in donors expressing specific HLA class I (Bw4-I80) may be less responsive under normal conditions. However, when a non-physiologic triggering signal is given to the cells (e.g., viral infections), the equilibrium that is maintaining tolerance could be disrupted and may become an important component of an efficient immune response (34). This mechanism could explain the association between the expression of *KIR3DS1* in conjunction with *HLA-B Bw4-I80* in patients with chronic HIV-1 infection and a slower progression to AIDS.

KIR3DS1 associates DAP12 ITAM-bearing molecules in its cytoplasmic tail to enable signal transduction. Surprisingly, in recent years, different studies showed that ITAMs can also generate an inhibitory signal in addition to the activating ones. In particular, the same ITAM-coupled receptors can generate both positive and negative signals (49–51). The molecular basis of this dual function is not well understood at the present; however, it has been suggested that the avidity of receptor ligation may define the nature of response. In this regard, one may speculate that the peptide in the HLA-Bw4-I80 groove could determine change of affinity of KIR3DS1 ligation. Thus, healthy self peptides could mediate a low-affinity, tolerogenic signal, whereas viral peptides (i.e., HIV derived) would allow high-affinity activating signals. This hypothesis would be in line with the protective effect exerted by the combined presence of *KIR3DS1* and *HLA-B Bw4-I80* in patients with chronic HIV-1 infection.

All these considerations are consistent with an important role of KIR3DS1 in the control of viral infections (8, 30), primarily in post-transplantation settings (52). Since transplantation from donors displaying NK-cell alloreactivity and expressing *KIR2DS1* and/or *KIR3DS1* has been associated with a reduced risk of no relapse mortality, that is largely infection related, and with significantly better event-free survival (38, 53), our results could further improve the selection of the most suitable donor, taking into account the expression of not only *KIR3DS1* but also the self HLA-B allotype expressed.

## Ethics Statement

Buffy coats from healthy donors were obtained from the Immunohematology and Transfusion Center of the S. Martino Hospital (Genova, Italy). Approval was obtained by the ethical committee of IRCCS S. Martino-IST (39/2012) of Genova (Italy). Informed consent was provided according to the Declaration of Helsinki.

## Author Contributions

SC and SS designed and performed research, interpreted data, and wrote the paper; MF performed research, interpreted data, and wrote the paper; MB, CA, LG, and MM performed research and analyzed data; LM interpreted and critically revised the paper; and AM interpreted, critically revised data, and wrote the paper.

## Conflict of Interest Statement

AM is founder and shareholder of Innate-Pharma (Marseille, France). The remaining authors have no conflicting financial interests.

## References

[1] Caligiuri MA. Human natural killer cells. Blood (2008) 112(3):461–9. 10.1182/blood-2007-09-077438 18650461 PMC2481557

[2] Vivier E Raulet DH Moretta A Caligiuri MA Zitvogel L Lanier LL Innate or adaptive immunity? The example of natural killer cells. Science (2011) 331(6013):44–9. 10.1126/science.1198687 21212348 PMC3089969

[3] Parham P. MHC class I molecules and KIRs in human history, health and survival. Nat Rev Immunol (2005) 5(3):201–14. 10.1038/nri1570 15719024

[4] Moretta A Bottino C Vitale M Pende D Biassoni R Mingari MC Receptors for HLA class-I molecules in human natural killer cells. Annu Rev Immunol (1996) 14:619–48. 10.1146/annurev.immunol.14.1.619 8717527

[5] Moretta A Sivori S Vitale M Pende D Morelli L Augugliaro R Existence of both inhibitory (p58) and activatory (p50) receptors for HLA-C molecules in human natural killer cells. J Exp Med (1995) 182(3):875–84. 10.1084/jem.182.3.875 7650491 PMC2192157

[6] Tomasello E Olcese L Vely F Geourgeon C Blery M Moqrich A Gene structure, expression pattern, and biological activity of mouse killer cell activating receptor-associated protein (KARAP)/DAP-12. J Biol Chem (1998) 273(51):34115–9. 10.1074/jbc.273.51.34115 9852069

[7] Parham P Norman PJ Abi-Rached L Guethlein LA. Variable NK cell receptors exemplified by human KIR3DL1/S1. J Immunol (2011) 187(1):11–9. 10.4049/jimmunol.0902332 21690332 PMC3223120

[8] Korner C Altfeld M. Role of KIR3DS1 in human diseases. Front Immunol (2012) 3:326. 10.3389/fimmu.2012.00326 23125843 PMC3485674

[9] Cella M Longo A Ferrara GB Strominger JL Colonna M. NK3-specific natural killer cells are selectively inhibited by Bw4-positive HLA alleles with isoleucine 80. J Exp Med (1994) 180(4):1235–42. 10.1084/jem.180.4.1235 7931060 PMC2191670

[10] Stern M Ruggeri L Capanni M Mancusi A Velardi A. Human leukocyte antigens A23, A24, and A32 but not A25 are ligands for KIR3DL1. Blood (2008) 112(3):708–10. 10.1182/blood-2008-02-137521 18502829

[11] Martin MP Gao X Lee JH Nelson GW Detels R Goedert JJ Epistatic interaction between KIR3DS1 and HLA-B delays the progression to AIDS. Nat Genet (2002) 31(4):429–34. 10.1038/ng934 12134147

[12] Alter G Martin MP Teigen N Carr WH Suscovich TJ Schneidewind A Differential natural killer cell-mediated inhibition of HIV-1 replication based on distinct KIR/HLA subtypes. J Exp Med (2007) 204(12):3027–36. 10.1084/jem.20070695 18025129 PMC2118524

[13] Alter G Rihn S Walter K Nolting A Martin M Rosenberg ES HLA class I subtype-dependent expansion of KIR3DS1+ and KIR3DL1+ NK cells during acute human immunodeficiency virus type 1 infection. J Virol (2009) 83(13):6798–805. 10.1128/JVI.00256-09 19386717 PMC2698561

[14] Carr WH Rosen DB Arase H Nixon DF Michaelsson J Lanier LL. Cutting edge: KIR3DS1, a gene implicated in resistance to progression to AIDS, encodes a DAP12-associated receptor expressed on NK cells that triggers NK cell activation. J Immunol (2007) 178(2):647–51. 10.4049/jimmunol.178.2.647 17202323 PMC2561215

[15] O’Connor GM Vivian JP Gostick E Pymm P Lafont BA Price DA Peptide-dependent recognition of HLA-B*57:01 by KIR3DS1. J Virol (2015) 89(10):5213–21. 10.1128/JVI.03586-14 25740999 PMC4442525

[16] Garcia-Beltran WF Holzemer A Martrus G Chung AW Pacheco Y Simoneau CR Open conformers of HLA-F are high-affinity ligands of the activating NK-cell receptor KIR3DS1. Nat Immunol (2016) 17(9):1067–74. 10.1038/ni.3513 27455421 PMC4992421

[17] Burian A Wang KL Finton KA Lee N Ishitani A Strong RK HLA-F and MHC-I open conformers bind natural killer cell Ig-like receptor KIR3DS1. PLoS One (2016) 11(9):e0163297. 10.1371/journal.pone.0163297 27649529 PMC5029895

[18] Lopez-Vazquez A Rodrigo L Martinez-Borra J Perez R Rodriguez M Fdez-Morera JL Protective effect of the HLA-Bw4I80 epitope and the killer cell immunoglobulin-like receptor 3DS1 gene against the development of hepatocellular carcinoma in patients with hepatitis C virus infection. J Infect Dis (2005) 192(1):162–5. 10.1086/430351 15942906

[19] Alicata C Pende D Meazza R Canevali P Loiacono F Bertaina A Hematopoietic stem cell transplantation: improving alloreactive Bw4 donor selection by genotyping codon 86 of KIR3DL1/S1. Eur J Immunol (2016) 46(6):1511–7. 10.1002/eji.201546236 26990677 PMC5065926

[20] Jiang W Johnson C Jayaraman J Simecek N Noble J Moffatt MF Copy number variation leads to considerable diversity for B but not A haplotypes of the human KIR genes encoding NK cell receptors. Genome Res (2012) 22(10):1845–54. 10.1101/gr.137976.112 22948769 PMC3460180

[21] Moretta A Tambussi G Bottino C Tripodi G Merli A Ciccone E A novel surface antigen expressed by a subset of human CD3- CD16+ natural killer cells. Role in cell activation and regulation of cytolytic function. J Exp Med (1990) 171(3):695–714. 10.1084/jem.171.3.695 2137855 PMC2187781

[22] Moretta A Vitale M Sivori S Bottino C Morelli L Augugliaro R Human natural killer cell receptors for HLA-class I molecules. Evidence that the Kp43 (CD94) molecule functions as receptor for HLA-B alleles. J Exp Med (1994) 180(2):545–55. 10.1084/jem.180.2.545 8046333 PMC2191622

[23] Pelak K Need AC Fellay J Shianna KV Feng S Urban TJ Copy number variation of KIR genes influences HIV-1 control. PLoS Biol (2011) 9(11):e1001208. 10.1371/journal.pbio.1001208 22140359 PMC3226550

[24] Foley BA De Santis D Van Beelen E Lathbury LJ Christiansen FT Witt CS. The reactivity of Bw4+ HLA-B and HLA-A alleles with KIR3DL1: implications for patient and donor suitability for haploidentical stem cell transplantations. Blood (2008) 112(2):435–43. 10.1182/blood-2008-01-132902 18385451

[25] Saunders PM Pymm P Pietra G Hughes VA Hitchen C O’Connor GM Killer cell immunoglobulin-like receptor 3DL1 polymorphism defines distinct hierarchies of HLA class I recognition. J Exp Med (2016) 213(5):791–807. 10.1084/jem.20152023 27045007 PMC4854737

[26] Fauriat C Ivarsson MA Ljunggren HG Malmberg KJ Michaelsson J. Education of human natural killer cells by activating killer cell immunoglobulin-like receptors. Blood (2010) 115(6):1166–74. 10.1182/blood-2009-09-245746 19903900

[27] Pyo CW Wang R Vu Q Cereb N Yang SY Duh FM Recombinant structures expand and contract inter and intragenic diversification at the KIR locus. BMC Genomics (2013) 14:89. 10.1186/1471-2164-14-89 23394822 PMC3606631

[28] Beziat V Traherne JA Liu LL Jayaraman J Enqvist M Larsson S Influence of KIR gene copy number on natural killer cell education. Blood (2013) 121(23):4703–7. 10.1182/blood-2012-10-461442 23637128 PMC3674669

[29] Pando MJ Gardiner CM Gleimer M McQueen KL Parham P. The protein made from a common allele of KIR3DL1 (3DL1*004) is poorly expressed at cell surfaces due to substitution at positions 86 in Ig domain 0 and 182 in Ig domain 1.J Immunol (2003) 171(12):6640–9. 10.4049/jimmunol.171.12.6640 14662867

[30] Carrington M Alter G. Innate immune control of HIV. Cold Spring Harb Perspect Med (2012) 2(7):a007070. 10.1101/cshperspect.a007070 22762020 PMC3385945

[31] Vitale M Sivori S Pende D Augugliaro R Di Donato C Amoroso A Physical and functional independency of p70 and p58 natural killer (NK) cell receptors for HLA class I: their role in the definition of different groups of alloreactive NK cell clones. Proc Natl Acad Sci U S A (1996) 93(4):1453–7. 10.1073/pnas.93.4.1453 8643653 PMC39960

[32] Moretta A Poggi A Olive D Bottino C Fortis C Pantaleo G Selection and characterization of T-cell variants lacking molecules involved in T-cell activation (T3 T-cell receptor, T44, and T11): analysis of the functional relationship among different pathways of activation. Proc Natl Acad Sci U S A (1987) 84(6):1654–8. 10.1073/pnas.84.6.1654 2951735 PMC304495

[33] Lee N Geraghty DE HLA-F surface expression on B cell and monocyte cell lines is partially independent from tapasin and completely independent from TAP. J Immunol (2003) 171(10):5264–71. 10.4049/jimmunol.171.10.5264 14607927

[34] Ivarsson MA Michaelsson J Fauriat C. Activating killer cell Ig-like receptors in health and disease. Front Immunol (2014) 5:184. 10.3389/fimmu.2014.00184 24795726 PMC4001058

[35] Pende D Marcenaro S Falco M Martini S Bernardo ME Montagna D Anti-leukemia activity of alloreactive NK cells in KIR ligand-mismatched haploidentical HSCT for pediatric patients: evaluation of the functional role of activating KIR and redefinition of inhibitory KIR specificity. Blood (2009) 113(13):3119–29. 10.1182/blood-2008-06-164103 18945967

[36] Venstrom JM Gooley TA Spellman S Pring J Malkki M Dupont B Donor activating KIR3DS1 is associated with decreased acute GVHD in unrelated allogeneic hematopoietic stem cell transplantation. Blood (2010) 115(15):3162–5. 10.1182/blood-2009-08-236943 20124216 PMC2858471

[37] Verheyden S Schots R Duquet W Demanet C. A defined donor activating natural killer cell receptor genotype protects against leukemic relapse after related HLA-identical hematopoietic stem cell transplantation. Leukemia (2005) 19(8):1446–51. 10.1038/sj.leu.2403839 15973456

[38] Mancusi A Ruggeri L Urbani E Pierini A Massei MS Carotti A Haploidentical hematopoietic transplantation from KIR ligand-mismatched donors with activating KIRs reduces nonrelapse mortality. Blood (2015) 125(20):3173–82. 10.1182/blood-2014-09-599993 25769621

[39] Gabriel IH Sergeant R Szydlo R Apperley JF DeLavallade H Alsuliman A Interaction between KIR3DS1 and HLA-Bw4 predicts for progression-free survival after autologous stem cell transplantation in patients with multiple myeloma. Blood (2010) 116(12):2033–9. 10.1182/blood-2010-03-273706 20562327 PMC6143153

[40] Sivori S Carlomagno S Falco M Romeo E Moretta L Moretta A. Natural killer cells expressing the KIR2DS1-activating receptor efficiently kill T-cell blasts and dendritic cells: implications in haploidentical HSCT. Blood (2011) 117(16):4284–92. 10.1182/blood-2010-10-316125 21355085

[41] Matusali G Tchidjou HK Pontrelli G Bernardi S D’Ettorre G Vullo V Soluble ligands for the NKG2D receptor are released during HIV-1 infection and impair NKG2D expression and cytotoxicity of NK cells. FASEB J (2013) 27(6):2440–50. 10.1096/fj.12-223057 23395909

[42] Guo H Kumar P Moran TM Garcia-Sastre A Zhou Y Malarkannan S. The functional impairment of natural killer cells during influenza virus infection. Immunol Cell Biol (2009) 87(8):579–89. 10.1038/icb.2009.60 19721456 PMC2882241

[43] Sene D Levasseur F Abel M Lambert M Camous X Hernandez C Hepatitis C virus (HCV) evades NKG2D-dependent NK cell responses through NS5A-mediated imbalance of inflammatory cytokines. PLoS Pathog (2010) 6(11):e1001184. 10.1371/journal.ppat.1001184 21085608 PMC2978723

[44] Li Y Wang JJ Gao S Liu Q Bai J Zhao XQ Decreased peripheral natural killer cells activity in the immune activated stage of chronic hepatitis B. PLoS One (2014) 9(2):e86927. 10.1371/journal.pone.0086927 24520324 PMC3919705

[45] Cerboni C Neri F Casartelli N Zingoni A Cosman D Rossi P Human immunodeficiency virus 1 Nef protein downmodulates the ligands of the activating receptor NKG2D and inhibits natural killer cell-mediated cytotoxicity. J Gen Virol (2007) 88(Pt 1):242–50. 10.1099/vir.0.82125-0 17170457

[46] Raulet DH. Roles of the NKG2D immunoreceptor and its ligands. Nat Rev Immunol (2003) 3(10):781–90. 10.1038/nri1199 14523385

[47] Fogli M Mavilio D Brunetta E Varchetta S Ata K Roby G Lysis of endogenously infected CD4+ T cell blasts by rIL-2 activated autologous natural killer cells from HIV-infected viremic individuals. PLoS Pathog (2008) 4(7):e1000101. 10.1371/journal.ppat.1000101 18617991 PMC2438610

[48] Pradeu T Jaeger S Vivier E. The speed of change: towards a discontinuity theory of immunity? Nat Rev Immunol (2013) 13(10):764–9. 10.1038/nri3521 23995627

[49] Pinheiro da Silva F Aloulou M Benhamou M Monteiro RC. Inhibitory ITAMs: a matter of life and death. Trends Immunol (2008) 29(8):366–73. 10.1016/j.it.2008.05.001 18602341

[50] Ivashkiv LB Cross-regulation of signaling by ITAM-associated receptors. Nat Immunol (2009) 10(4):340–7. 10.1038/ni.1706 19295630 PMC2753670

[51] Turnbull IR Colonna M. Activating and inhibitory functions of DAP12. Nat Rev Immunol (2007) 7(2):155–61. 10.1038/nri2014 17220916

[52] Stern M Hadaya K Honger G Martin PY Steiger J Hess C Telomeric rather than centromeric activating KIR genes protect from cytomegalovirus infection after kidney transplantation. Am J Transplant (2011) 11(6):1302–7. 10.1111/j.1600-6143.2011.03516.x 21486386

[53] Venstrom JM Pittari G Gooley TA Chewning JH Spellman S Haagenson M HLA-C-dependent prevention of leukemia relapse by donor activating KIR2DS1. N Engl J Med (2012) 367(9):805–16. 10.1056/NEJMoa1200503 22931314 PMC3767478

