# Supplementary material for: KIR3DS1-Mediated Recognition of HLA-*B51: Modulation of KIR3DS1 Responsiveness by Self HLA-B Allotypes and Effect on NK Cell Licensing
Source: Front Immunol. 2017 May 26;8:581. doi: 10.3389/fimmu.2017.00581 (PMC5445109; doi:10.3389/fimmu.2017.00581)
Supplement: Supplementary file 1 [file Presentation_1.PDF]

Supplementary Figure 1

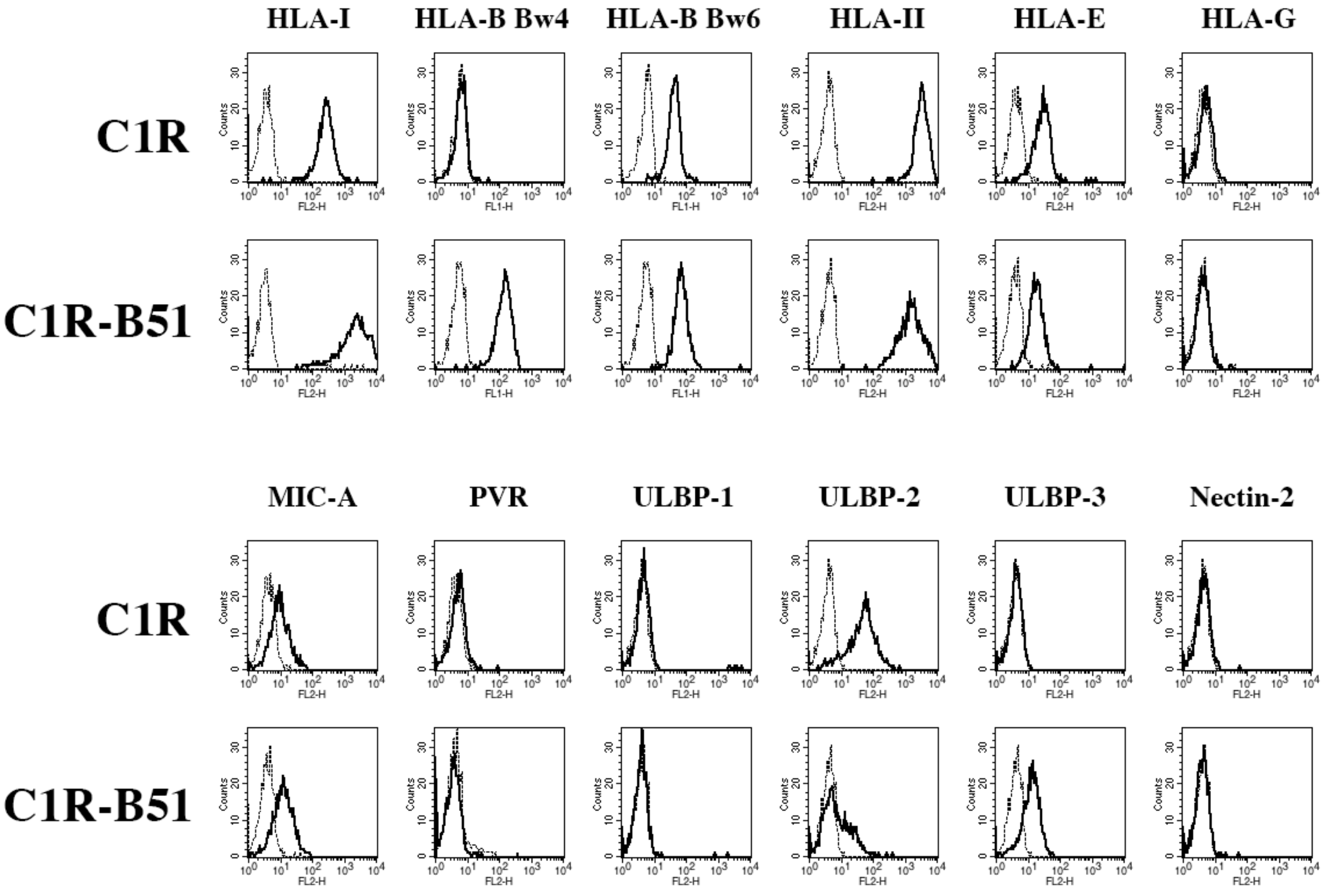

Supplemetary Figure 1. Surface phenotype of C1R and C1R-B51 cell lines.
Surface expression of HLA molecules and ligands for different non HLA-specific activating NK receptors on C1R and C1R-B51 cell lines were tested by cytofluorimetric analysis.

Supplemental Figure 2

	KIR gene repertoire																				KIR-L					
Donor	3DL3	2DS2	2DL2	2DL3	2DL5B	2DS3/S5	2DP1	2DL1	3DP1	RS	2DL4	3DL1	3DS1	2DL5A	2DS3/S5	2DS1	2DS4	3DL2	Cen	Tel	C1	C2	Bw4T	Bw4I	A Bw4	Bw4 $^{D77/T80}$
K40												C			S5				A/A	A/B						
P61						S3						C			S3				A/B	A/B						
V30												C			S5				A/B	A/B						
J56						S3 or S5						C			S3 or S5				B/B	A/B						
GL97												T			S5				A/A	A/B						
J47												C/T			S3				A/B	A/B						
J19						S3						C			S3				A/B	A/B						
J37												T			S5				A/B	A/B						
GL98												T			S5				A/A	A/B						
J102												C			S5				A/A	A/B						
GL118												C			S5				A/A	A/B						
J85												C			S5				A/A	A/B						
J66						S3 S5						C							A/B	A/B						
J67												C			S3				A/B	A/B						
GL141												C			S5				A/A	A/B						
GL143												C			S5				A/A	A/B						
U59						S3						C			S3				A/B	A/B						
U73												C			S5				A/B	A/B						
GL144												C			S5				A/B	A/B						
U105						S3						C			S3				A/B	A/B						
U108						S3 or S5						C			S3 or S5				A/B	A/B						
J13												C			S5				A/A	A/B						
J76						S3						C			S3				B/B	A/B						
J71						S3 or S5						C			S3 or S5				A/B	A/B						
GL115												C			S5				A/A	A/B						
U58												C			S5				A/A	A/B						
U116						S3 or S5						C			S3 or S5				B/B	A/B						
J38						S3 or S5						C			S3 or S5				A/B	A/B						
GL110												C			S5				A/B	A/B						
J75												C			S5				A/A	A/B						
T28						S3 or S5									S3 or S5				A/B	B/B						
T34						S3 or S5									S3 or S5				A/B	B/B						
S4															S5				A/A	B/B						
Z125															S5				A/B	B/B						
P230						S3 or S5									S3 or S5				A/B	B/B						
T33						S5									S5				A/B	B/B						
T42						S3 or S5									S3 or S5				A/B	B/B						
SICA												C			S5				A/A	A/B						
K9						S3 or S5						C			S3 or S5				B/B	A/B						
K7						S3						T			S3				A/B	A/B						

Supplementary Figure 2. Donor *KIR* and *KIR-L* genotypes.
The donor *KIR* genotypes were analyzed for the presence (grey boxes) or absence (white boxes) of all the *KIR* loci. The gene order was based on published *KIR* haplotype sequences. S3 and S5, inserted in the boxes, indicated the presence of *KIR2DS3* and *KIR2DS5* respectively. In donors typed *KIR2DS3*^{pos}, *KIR2DS5*^{pos}, *KIR2DL5A*^{pos}, and *KIR2DL5B*^{pos} were not possible determined the correct order of these two activating KIRs, thus, in these genotypes *S3 or S5* were assigned at both centromeric and telomeric regions. In *KIR3DL1* boxes the SNP at codon 186 that characterized the alleles coding for surface receptors (C) or coding for polypeptides retained into the cell (T) are reported. RS stated recombination hotspot site located between centromeric and telomeric regions. Analysis of centromeric and telomeric regions are reported.

Supplementary Figure 3

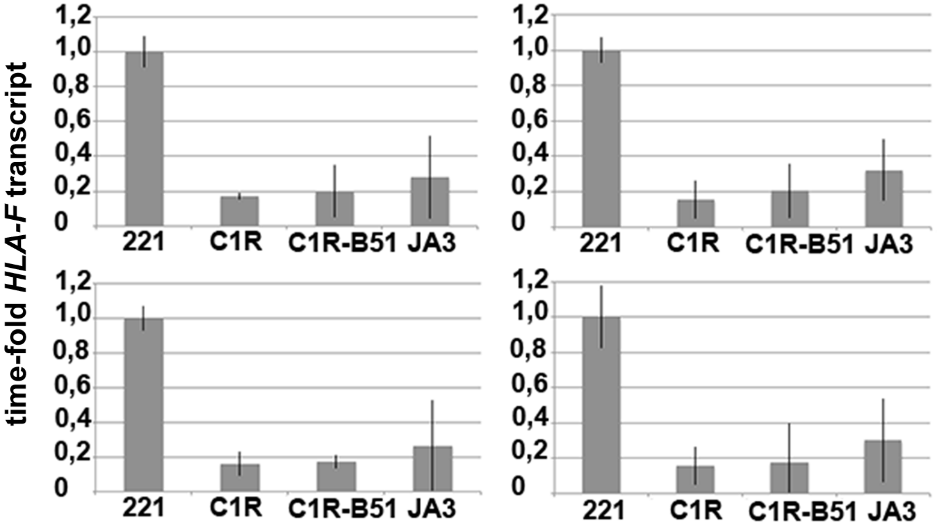

Supplementary Figure 3. Analysis of *HLA-F* transcript.

Data obtained in four independent experiments are shown. Each bar represents *HLA-F* mRNA amount, normalized to *GAPDH* transcript, detected in the analyzed cell line. LCL 721.221 cell line (used as reference) represents the positive control whereas JA3 was chose as negative control. In each experiment the indicated cell lines were analyzed in triplicate. Means and standard deviations are reported.
